# Immunoglobulins to mitigate paraneoplastic Lambert Eaton Myasthenic Syndrome under checkpoint inhibition in Merkel cell carcinoma

**DOI:** 10.1186/s42466-020-00099-5

**Published:** 2020-12-09

**Authors:** Maike F. Dohrn, Ulrike Schöne, Charlotte Küppers, Deborah Christen, Jörg B. Schulz, Burkhard Gess, Simone Tauber

**Affiliations:** 1 Department of Neurology, Medical Faculty of the RWTH Aachen University, Aachen, Germany; 2 Dr. John T. Macdonald Foundation, Department of Human Genetics and John P. Hussman Institute for Human Genomics, University of Miami, Miller School of Medicine, Miami, Florida USA; 3 Department of Oncology and Hematology, Medical Faculty of the RWTH Aachen University, Aachen, Germany

## Abstract

Lambert-Eaton myasthenic syndrome (LEMS) is a rare, autoimmune or paraneoplastic condition characterized by muscle weakness and fatigability. In cancer therapy, immune checkpoint inhibitors (ICI) sensitize the immune system for tumor antigens.

We report a 62-year-old, female patient with paraneoplastic LEMS as first manifestation of Merkel cell carcinoma. Under avelumab, the LEMS exacerbated with worsening of limb weakness and a severely reduced vital capacity (< 1 l). To treat this immunological side effect, we added a regimen with intravenous immunoglobulins. Hereby, the LEMS improved significantly. As we were able to continue the cancer treatment, the Merkel cell carcinoma has been in remission so far.

This is the first description of paraneoplastic LEMS, avelumab, and Merkel cell carcinoma. We conclude that immunoglobulins are an option to control an ICI-associated deterioration of paraneoplastic symptoms.

## Case

**Fig. 1:**
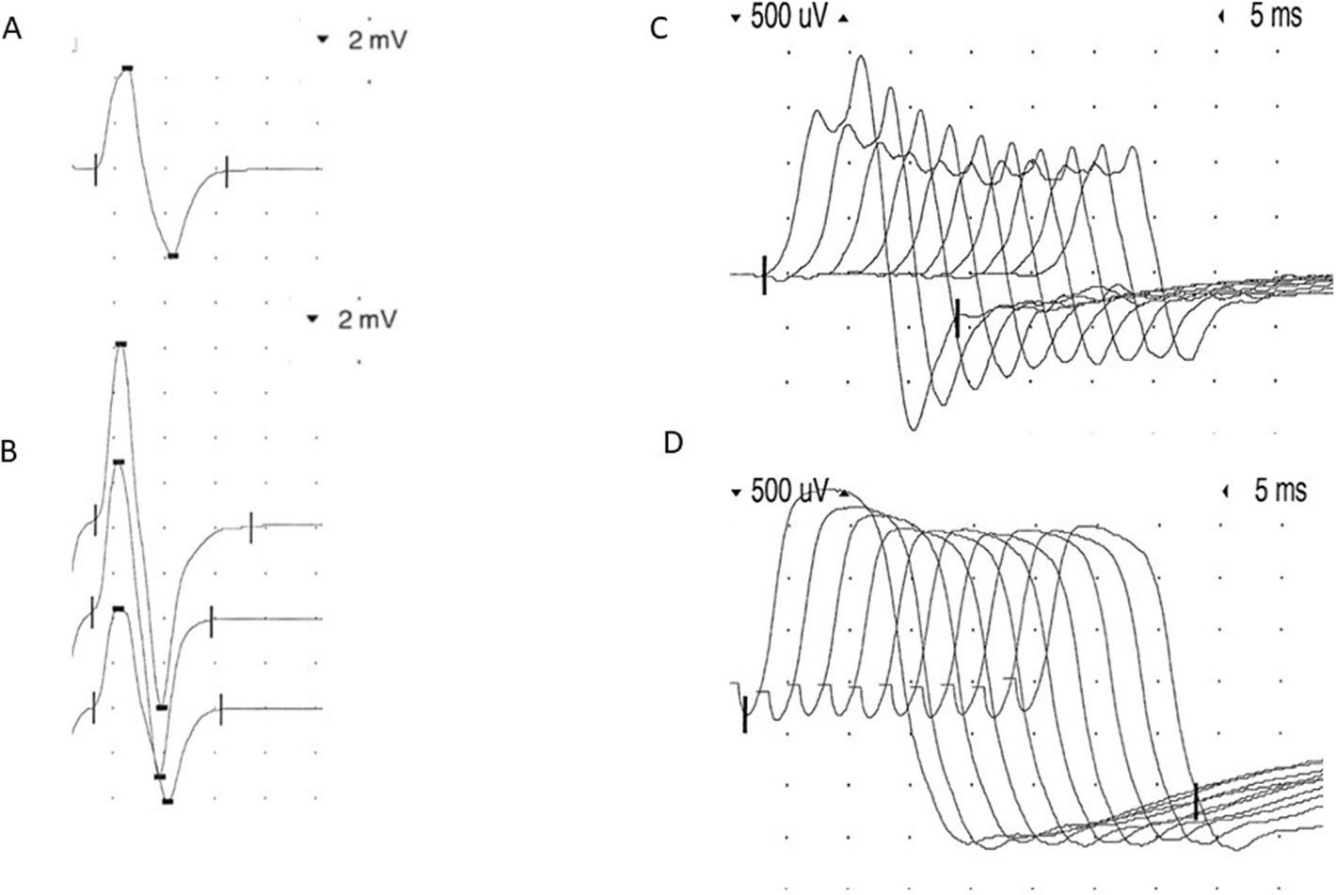
Neurophysiological examinations. **a** Compound muscle action potential (CMAP) of the left abductor digiti minimi muscle (8.4 mV). **b** Significant increment (16.2 mV) following tetanic stimulation. **c** 43% decrement under 3 Hz repetitive stimulation of the left anconeus muscle. **d** 19% decrement of the right trapezius muscle (at first visit only)

A 62-year-old female patient presented with bilateral ptosis, dysphagia, and combined distal and proximal muscle weakness and fatigability. The clinical examination revealed a positive Simpson-test, hand muscle weakness, and an impaired proximal endurance. The 3 Hz-repetitive stimulation showed a significant decrement of compound muscle action potentials (CMAP) of the left trapezius and both anconeus muscles. PQ-type VGCC antibodies were elevated in serum by 387.6 pmol/l (< 40), while antibodies against the acetylcholine receptor were negative. We began a symptomatic treatment of the Lambert-Eaton myasthenic syndrome (LEMS) with 3,4-diaminopyridine and pyridostigmine. As 50% of LEMS cases arise in a paraneoplastic context [1], a PET-CT revealed inguinal lymph nodes suspicious for malignancy. A biopsy disclosed lymphogenic metastases of Merkel cell carcinoma, the primary origin of which remained unknown. The patient received avelumab, a recently approved immune checkpoint inhibitor (ICI) [2], which led to tumor remission. To enhance the immune response, avelumab targets the programmed cell death ligand (PD-L1), which is known to be upregulated by tumor cells to escape the recognition of T cells. In this patient, ICI treatment led to a significant worsening of limb weakness requiring the use of a walker, and a severely reduced vital capacity (< 1 l). The risks of both receiving and withdrawing avelumab were addressed in an interdisciplinary oncological board and discussed with the patient. Considering the severity of the underlying malignancy, ICI treatment could not be halted for good. For the same reason, cell-depleting immunosuppressants such as rituximab were not our first choice of treatment, especially as the combination of such drugs with avelumab has never been examined. Plasmapheresis was discussed as an option, but not preferred considering that a long-term therapy was needed. As the patient already hat type 2 diabetes mellitus and osteoporosis, we aimed at avoiding a repetitive or long-term steroid treatment as well. An immediate intervention with intravenous immunoglobulins in a dosage of 2 g/kg body weight over five consecutive days enabled a subjective improvement of symptoms. We continued the immunoglobulin treatment in an intermittent dosage of 1 g/kg body weight every four weeks, which was well tolerated. The patient reported a dynamic worsening of symptoms in the last week before and a subjective improvement following immunoglobulin treatment. After six-months, an improvement in proximal strength enabled her to walk up to 500 m. The endurance tests of both arms and legs had normalized. The distal muscle strength was still impaired, and the Simpson test remained positive. The vital capacity was 1.5 l, and the Besinger’s score had declined from 6 to 4 points. The former decrement of the left trapezius muscle was no longer reproducible. Following tetanic stimulation, there was a significant CMAP increment of the abductor digiti minimi muscles on both sides (Fig. 1a, b). An anconeus muscle decrement became apparent in the 3 Hz repetitive stimulation of both radial nerves (Fig. 1c). The Merkel cell carcinoma has so far been in complete remission.

## Discussion

Enabling the immune system to resist elaborate tumor escape strategies such as PD-L1 expression, the concept of immune checkpoint inhibition has won the Nobel prize in 2018. Antagonists against this or other ligands and receptors, however, can lead to the collateral offense of other targets including the pre-synaptic calcium channel VGCC that structurally resembles certain tumor antigens [3, 4]. A paraneoplastic LEMS can precede the tumor diagnosis by several years [1]. There are no trial-based data on using ICI drugs in cases of pre-existing paraneoplastic syndromes [5], especially if the tumor stage prevents other alternatives.

This patient is the first case receiving avelumab despite having a paraneoplastic LEMS. Other authors described four patients with avelumab-treated thymoma developing myositis [6] and another patient with small lung cell carcinoma developing LEMS while being treated with nivolumab [7].

With the patient’s yet metastasized malignancy on the one and the potentially life-threatening LEMS on the other hand, we had to outweigh the respective risks and chose to add intravenous immunoglobulins to the previous ICI treatment. In the six-months observation interval, the patient thereby improved significantly. We conclude that immunoglobulins are a conceivable treatment option to control an ICI-associated deterioration of paraneoplastic symptoms.

## Data Availability

Not applicable.

## Abbreviations

CMAP: Compound muscle action potential
ICI: Immune checkpoint inhibition
LEMS: Lambert Eaton Myasthenic Syndrome
PD-L1: Programmed cell death ligand 1
VGCC: Voltage-gated calcium channel
